# Retro-pancreatic pull-through reconstruction of the hypoplastic portal vein using the autologous mesosystemic shunt vessel in adult living donor liver transplantation: a case report

**DOI:** 10.1186/s40792-024-01863-4

**Published:** 2024-04-02

**Authors:** Shinsuke Sugenoya, Atsuyoshi Mita, Akira Shimizu, Yasunari Ohno, Koji Kubota, Yuichi Masuda, Tsuyoshi Notake, Yuji Soejima

**Affiliations:** Division of Gastroenterological, Hepato-Biliary-Pancreatic, Transplantation and Pediatric Surgery, Department of Surgery, Shinshu University School of Medicine, 3-1-1 Asahi, Matsumoto, 390-8621 Japan

**Keywords:** Retro-pancreatic pull-through reconstruction, Hypoplastic portal vein, Autologous mesosystemic shunt vessel, Adult living donor liver transplantation, Biliary atresia

## Abstract

**Background:**

In liver transplant patients with hypoplastic portal vein (PV), when the narrowed segment is extended too deep into the dorsal side of the pancreas, it is difficult and dangerous to reconstruct the interposition graft from the upper part of the pancreas. Herein, we present a case of PV reconstruction with the autologous mesosystemic shunt vessel from the caudal side of the pancreas in a situation where the narrowed PV was deep, and we discuss the technical details.

**Case presentation:**

A 25-year-old woman presented with cholestatic liver cirrhosis due to biliary atresia after Kasai procedure. Since her jaundice progressed, she was referred to our hospital for liver transplantation. Laboratory tests showed that her total bilirubin was elevated to 7.6 mg/dL. The Model for End-Stage Liver Disease score was 18, and the Child–Pugh score was 9 (Grade B). She underwent living donor liver transplantation (LDLT) using a right hemi-liver graft procured from her 54-year-old mother. The conventional approach from the cephalad side to the superior mesenteric vein (SMV) and splenic vein (SpV) confluence behind the pancreas was extremely difficult in this case because the confluence of SMV and SpV was close to the lower edge of the pancreas. Therefore, we decided to perform PV reconstruction from the caudal side. The main trunk of PV was documented as narrow (5 mm in diameter), for which retro-pancreatic pull-through PV reconstruction was successfully performed using her own mesosystemic shunt vessel. A contrast computed tomography (CT) scan was performed on postoperative day 5 because of an elevation of D-dimer and found a partial thrombus in the left pulmonary artery, as well as in the PV and left renal vein. Thereafter, thrombolytic therapy with low-molecular-weight heparin was started immediately and switched to a direct oral anticoagulant. The follow-up CT taken 3 months after liver transplantation revealed a patent PV without thrombus; therefore, anticoagulant therapy was discontinued. Currently, the patient has been well and active with a patent PV without anticoagulant therapy for 3 years after LDLT.

**Conclusions:**

Retro-pancreatic pull-through reconstruction of the hypoplastic PV is a feasible and effective method when conventional reconstruction is not indicated.

## Background

In patients with biliary atresia, the portal vein (PV) is often highly hardened/narrowed or accompanied by PV thrombosis due to frequent surgical procedures and inflammation in association with cholangitis. Ensuring sufficient PV blood flow after liver transplantation (LT) is important for avoiding graft failure [1], but complications by caliber mismatch, PV twist, and thrombosis are likely to occur, leading to graft dysfunction [2–4]. Therefore, various anastomosis/reconstruction methods have been proposed so far [1, 5–10].

It is technically difficult to perform PV reconstruction from the cephalad side of the pancreas in cases where the narrowed PV is located deep in the dorsal side of the pancreas. Herein, we present a case in which the autologous mesosystemic shunt vessel (gonadal vein) dilated due to PV stenosis for portal hypertension was used as an interstitial graft and reconstructed through the dorsal side of the pancreas.

## Case presentation

**Fig. 1:**
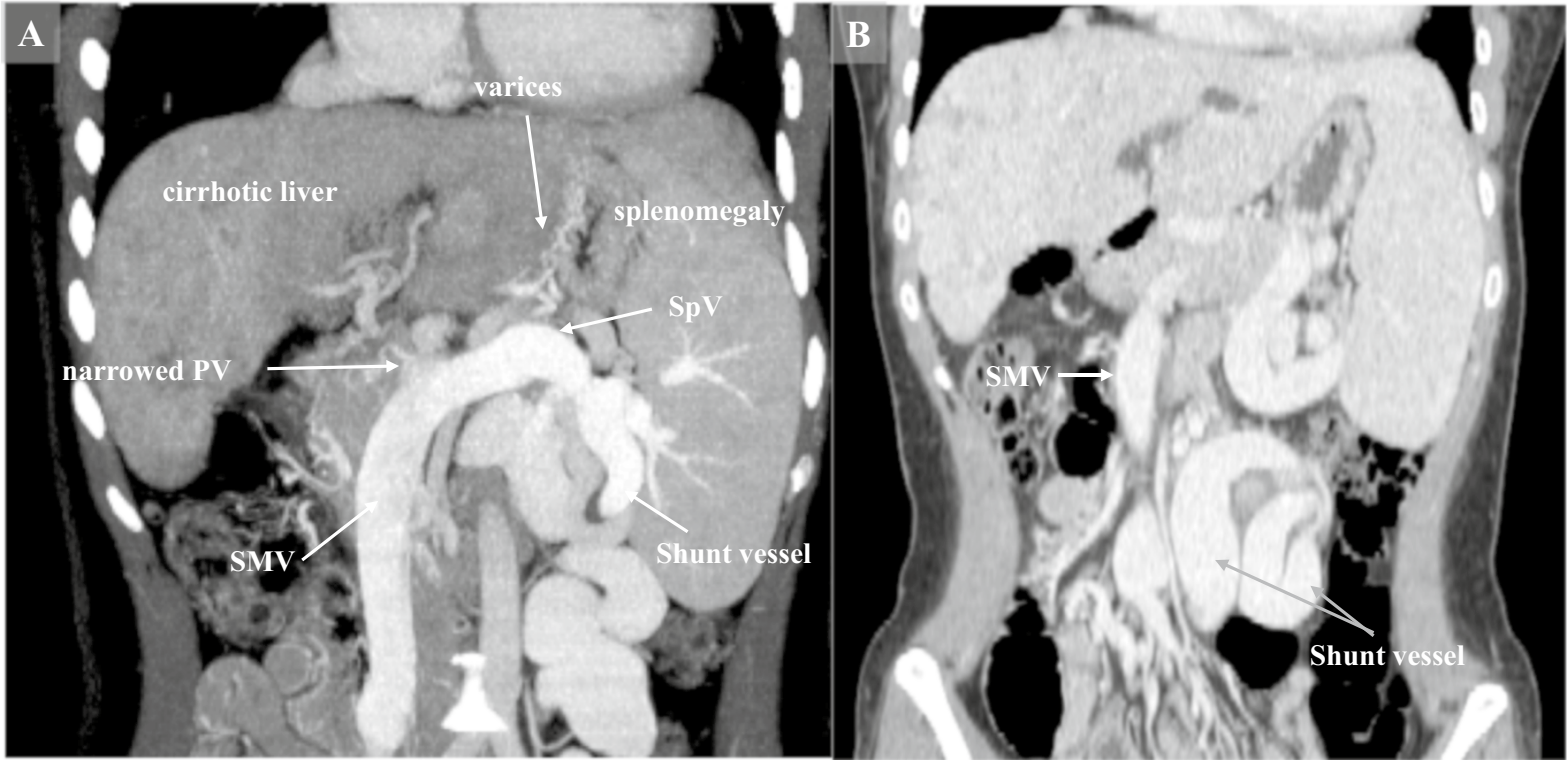
**a** Preoperative enhanced abdominal CT scans in coronary view showed the hypoplastic portal vein (PV) in 5 mm of diameter and dilated collateral vessels, such as splenic vein, superior mesenteric vein (SMV), and a shunt vessel. The liver exhibited cirrhotic changes accompanied by splenomegaly. **b** The gonadal vein was increased in length by 20 cm and in thickness by 2 cm and formed the mesosystemic shunt vessel between the SMV and left renal vein

A 25-year-old woman presented with cholestatic liver cirrhosis due to biliary atresia following a Kasai procedure 47 days after childbirth. Twenty-five years after the operation, jaundice appeared, and she was referred to our hospital for LT. Laboratory tests showed a total bilirubin level of 13.94 mg/dL, platelet count of 13.5 × 10^4^/µL, and prothrombin time-international normalized ratio of 1.18 (Table 1). The Model for End-Stage Liver Disease score was 18, and the Child–Pugh score was 9 (Grade B). The patient had no history of smoking, alcohol, or use of recreational drugs. Preoperative contrast computed tomography (CT) scan revealed an enlarged cirrhotic liver, esophagogastric varices, narrowed PV of 5 mm in diameter, and splenomegaly (Fig. 1a). The mesosystemic shunt vessel (gonadal vein) was increased in length by 20 cm and in thickness by 2 cm between the superior mesenteric vein (SMV) and left renal vein (Fig. 1b). Thrombosis was not found in the PV. She underwent living donor LT (LDLT) using a right hemi-liver graft procured from her 54-year-old mother.

**Table 1:** Laboratory data

WBC	3.57	× 10^3^/µL	Cre	0.47	mg/dL
Hb	12.6	g/dL	AST	169	U/L
HCT	37.7	%	ALT	103	U/L
PLT	13.5	× 10^4^/µL	T-bil	13.94	mg/dL
			Na	140	mmol/L
ALB	3.3	g/dL			
BUN	7.4	mg/dL	PT-INR	1.18	

*WBC* white blood cells, *Hb* hemoglobin, *HCT* hematocrit, *PLT* platelet, *ALB* albumin, *BUN* blood urea nitrogen, *Cre* creatinine, *AST* aspartate aminotransferase, *ALT* alanine aminotransferase, *T-bil* total bilirubin, *Na* sodium, *PT-INR* prothrombin time-international normalized ratio

## Surgical procedures

**Fig. 2:**
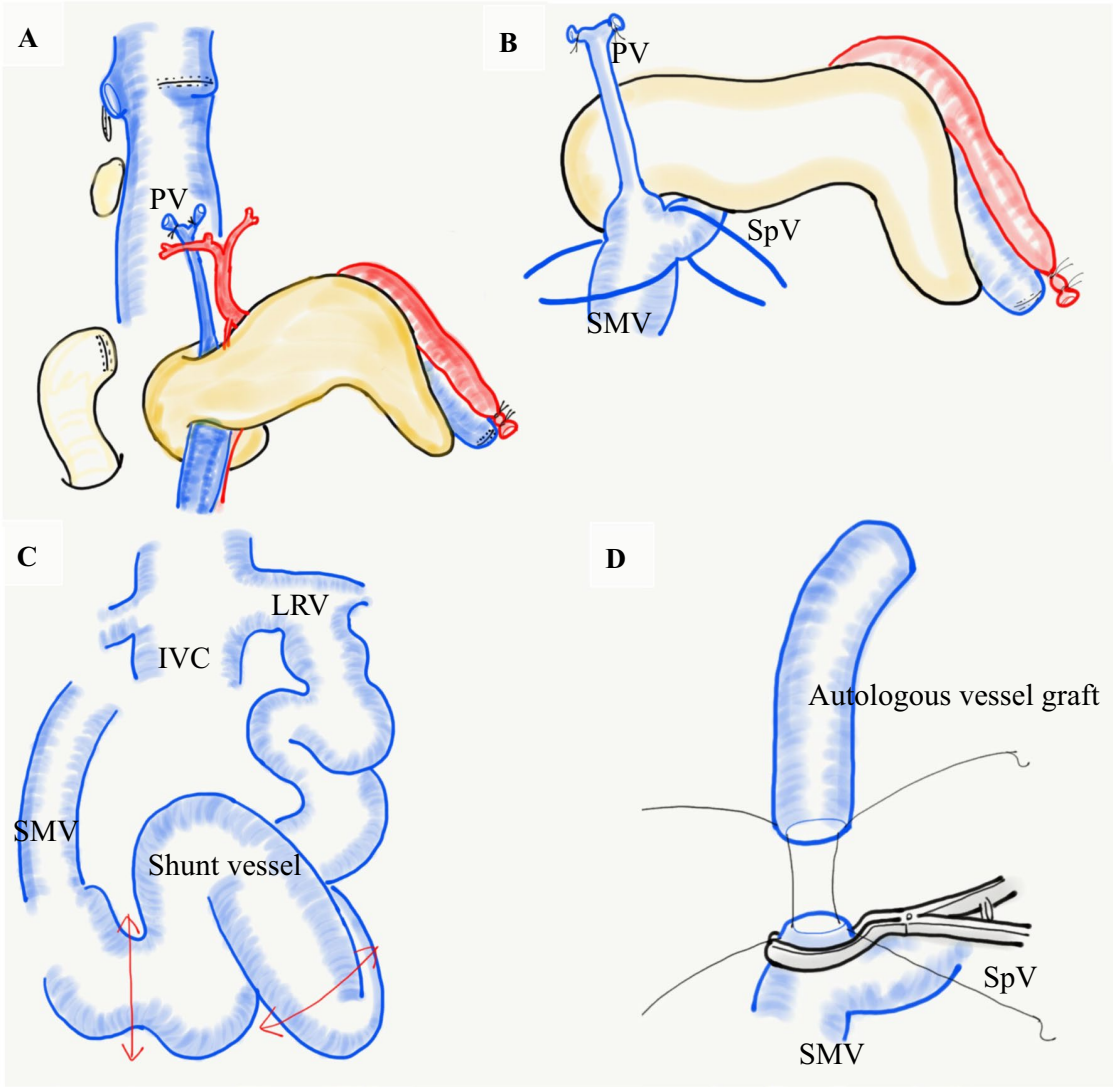
Illustrated technical highlights. **a** The portal vein (PV) was isolated by dividing branches from the pancreas and was drawn from the superior to the inferior border of the pancreas. **b** Subsequently, the PV was trimmed. The superior mesenteric vein (SMV) and the splenic vein (SpV) were taped on the caudal side of the pancreas. **c** The dilated shunt vessel connecting between the SMV and the left renal vein was isolated and used as an autologous vessel graft. **d** The autologous vessel graft was anastomosed to the confluence of SMV and SpV

After total hepatectomy and splenectomy, the main trunk of the PV was cut, which revealed insufficient portal flow due to hypoplastic PV. We decided to remove the narrowed segment and reconstruct the PV using an interposition graft. We procured an autologous mesosystemic shunt vessel flowing into the left renal vein with a diameter of 17 mm and length of 8 cm as an interposition graft (Fig. 2c).

**Fig. 3:**
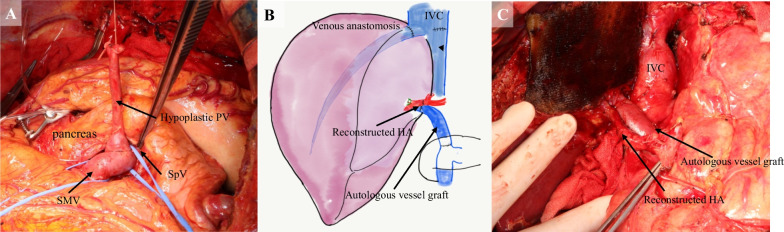
**a** Intraoperative image showed the hypoplastic portal vein (PV) trunk being drawn from the superior to the inferior border of the pancreas. It corresponds to Fig. 2b. SMV, superior mesenteric vein; SpV, splenic vein. **b** Schema after vessel reconstructions. The right hepatic vein of the liver graft was anastomosed to the stump of the right hepatic vein. Subsequently, the autologous vessel graft was anastomosed to the right branch of the PV in the liver graft. Arterial reconstruction was microscopically performed between the right hepatic artery in the liver graft and the right hepatic artery in the recipient. IVC, inferior vena cava. **c** The image after vessel reconstructions

However, the conventional approach from the cephalad side to the SMV and splenic vein (SpV) confluence behind the pancreas was extremely difficult in the present case. Therefore, we decided to perform PV reconstruction from the caudal side. The SMV was identified and taped on the caudal side of the pancreas, tunneled through the posterior surface of the pancreas (Fig. 2a), and the head of the pancreas was raised using a 6 Fr. atom tube. All the inflow branches from the pancreas flowing into the SMV and PV were ligated and dissected, and the main PV trunk was pulled out to the caudal side of the pancreas (Figs. 2b, 3a).

A narrow part of the PV trunk was removed, and one stump of the autologous vascular graft was continuously sutured to the confluence of SMV and SpV using 6-0 PDS-II (Ethicon Co., Tokyo, Japan) with continuous sutures (Fig. 2d). The autologous vascular graft was guided through the dorsal side of the pancreas to the upper edge of the pancreas.

Subsequently, the grafted liver was inserted, and the hepatic vein reconstruction was performed, followed by the PV reconstruction. The stump of the interposition graft was trimmed to an appropriate length and anastomosed to the graft PV using 6-0 PDS-II with continuous sutures (Fig. 3b, c). After reperfusion, the hepatic artery anastomosis and biliary reconstruction were performed as a regular sequence. The operative time was 1022 min, the blood loss was 5700 g, the cold ischemic time was 353 min, and the warm ischemic time was 55 min.

## Posttransplant follow-up

**Fig. 4:**
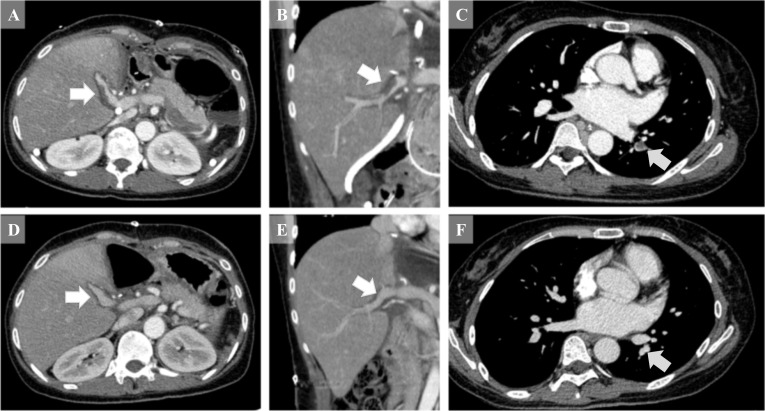
Partial thrombus in the portal (PV) and left renal veins revealed by computed tomography (CT). **a**–**c** Contrast-enhanced CT scans performed on postoperative day 5 revealed partial thrombus in the PV (white arrows) and the left pulmonary artery (grey arrow). **d**–**f** Contrast-enhanced CT revealed the disappearance of the thrombus in the PV (white arrows) and the left pulmonary artery (grey arrow) after 3 months

Postoperatively, no anticoagulant treatment was administered at first. The recipient’s vascular integrity was monitored serially using Doppler ultrasonography. A contrast CT scan was performed on postoperative day (POD) 5 because of an elevation of D-dimer and revealed partial thrombus in the PV (Fig. 4a, b), as well as in the left pulmonary artery (Fig. 4c) and left renal vein. A Doppler ultrasonography showed hepatophilic blood flow in the PV of the liver graft. Thrombolytic therapy with low-molecular-weight heparin was started immediately and switched to direct oral anticoagulant on POD 18. The recipient was discharged from our hospital and visited our outpatient clinic every 1–3 months. The follow-up contrast CT scan taken 3 months after LT revealed a patent PV (Fig. 4d, e) and pulmonary artery (Fig. 4f) without thrombus; therefore, anticoagulant therapy was discontinued. Her liver graft has functioned optimally 3 years after LDLT.

## Discussion

We successfully reconstructed the PV using the autologous mesosystemic shunt vessel by retro-pancreatic pull-through reconstruction method in the case of narrowed PV. Reconstruction can be challenging in a PV that is narrow or obstructed by thrombus in the setting of LDLT using an autologous vascular graft of insufficient length. Here, we describe an alternative procedure. In the present case, we adopted the above method due to the following reasons. First, the confluence of SMV and SpV was close to the lower edge of the pancreas, making it difficult to perform anastomosis from the upper edge of the pancreas. Second, the recipient had a dilated/tortuous ovarian vein suitable for grafting. Third, due to insufficient graft length as a jumping graft method, the narrowed PV was once pulled out from the lower edge of the pancreas to the caudal side (pullout method [3]), and the PV trunk was reconstructed with the interposition graft and then, anastomosed with the PV of the grafted liver through the dorsal side of the pancreas.

The pullout method, previously employed in a pediatric patient with PV obstruction [3], is dangerous for adult patients because collateral vessels usually develop around the SMV/SpV confluence. Mizuno et al. reported a similar method in three adult cases of PV thrombosis [7]. Technical tips of the method are as follows. It is necessary to carefully ligate and dissect the veins flowing into the PV on the dorsal side of the pancreas to completely free the PV stenosis from the pancreas. Bleeding from the retro-pancreatic area can be uncontrollable and disastrous in patients with portal hypertension.

When we procured autologous vein grafts, we usually select the internal jugular vein that could be easily harvested and has excellent graft quality [11]. A great saphenous vein [12], external iliac vein [7, 10], and a renal vein [13] have also been used as autologous vein grafts. An artificial blood vessel was used as an alternative for PV reconstruction without available autologous vein grafts [14]. The mesosystemic shunt vessel should be ligated to prevent the diversion of blood flow from the PV into the systemic circulation, as in the present case, enabling us to use it as an autologous vein graft. In addition, we supposed that using the mesosystemic shunt vessel provided an esthetic advantage to obtain a sufficient graft without any additional incisions in the present case.

For anticoagulant therapy, prophylactic measures might be necessary for this type of unconventional PV reconstruction. In the present case, a contrast CT scan taken 6 days postoperatively revealed a thrombosis of the left pulmonary artery, PV, and the collateral circulation to the left renal vein and SpV. We supposed that the pulmonary artery and the PV were thrombosed by a thrombus derived from either the left renal vein, SpV, or the stump of the shunt vessels. With the use of low-molecular-weight heparin, a follow-up ultrasonography revealed no expansion of the PV thrombus. Contrast-enhanced CT on PODs 13 and 20 revealed a reduction of the thrombus; therefore, anticoagulant therapy was switched to direct oral anticoagulant. Retrospectively, it is considered that the blockage of blood flow by ligation of the mesosystemic shunt vessel at the confluence to the SpV could prevent the growth of thrombus from the collateral circulation to the SpV.

The patient was discharged from our hospital on POD 143. Currently, the patient has been well and active with a patent PV without anticoagulant therapy at 3 years after LDLT.

## Conclusions

Retro-pancreatic pull-through reconstruction of the hypoplastic PV is a feasible and effective method when conventional reconstruction is not indicated.

## Data Availability

All data generated or analyzed during this study are included in this published article.

## Abbreviations

CT: Computed tomography
LDLT: Living donor liver transplantation
LT: Liver transplantation
POD: Postoperative day
PV: Portal vein
SMV: Superior mesenteric vein
SpV: Splenic vein
